# Winter temperature correlates with mtDNA genetic structure of yellow-necked mouse population in NE Poland

**DOI:** 10.1371/journal.pone.0216361

**Published:** 2019-05-08

**Authors:** Sylwia D. Czarnomska, Magdalena Niedziałkowska, Tomasz Borowik, Bogumiła Jędrzejewska

**Affiliations:** Mammal Research Institute, Polish Academy of Sciences, Białowieża, Poland; Ben-Gurion University of the Negev, ISRAEL

## Abstract

We analysed a fragment (247 bp) of cytochrome *b* of mitochondrial DNA sequenced using 353 samples of yellow-necked mice *Apodemus flavicollis* trapped in seven forests and along three woodlot transects in north-eastern Poland. Our aims were to identify the phylogeographic pattern and mtDNA structure of the population and to evaluate the role of environmental conditions in shaping the spatial pattern of mtDNA diversity. We found out that three European haplogroups occurred sympatrically in north-eastern Poland. Inferences based on mtDNA haplotype distribution and frequency defined five subpopulations. The mtDNA-based structure of mice significantly correlated with winter temperature: frequency of Haplogroup 1 was positively, and that of Haplogroup 3 negatively correlated to mean temperature of January in the year of trapping. Synthesis of the published pan-European data on the species phylogeography also showed that the possibly 'thermophilous' Haplogroup 1 has the westernmost occurrence, whereas the more 'cold-resistant' Haplogroup 3 occurs much further to north-east than the other haplogroups. The observed patter may be a byproduct of the tight coevolution with nuclear genes, as we have earlier found that − in mice population in NE Poland − the spatial pattern of nuclear DNA was best explained by January temperature. Alternatively, the observed association of mitochondrial genetic variation with temperature is possible to be adaptive as cytochrome *b* is involved in the process of ATP production via oxidative phosphorylation.

## Introduction

Historical events such as isolation in glacial refugia during the Last Glacial Maximum (LGM), landscape discontinuities created by ice sheets and recolonization routes at the end of Pleistocene are still detectable in genomes of contemporary living animals. It is commonly believed that during the glacial maxima the ranges of temperate and boreal species were restricted to Mediterranean refugia [1–4]. However, as some phylogenetic studies showed, there had to be more refugial areas, placed somewhere in the eastern part of the continent but their exact localization is still under debate [3, 5, 6].

Although some common patterns of the impact of Quaternary glaciations on species persistence were identified [1, 2], it seems that each species responded to changing climate differently, depending on their specific adaptations [7]. Most of the small mammal species studied so far exhibit similar spatial pattern, namely allopatric/parapatric distributions of lineages with relatively narrow secondary contact zones e.g. [4, 8–9]. Contrary to that, phylogeography of yellow-necked mouse *Apodemus flavicollis* (Melchior, 1834) one of the most common species of forest small mammals of temperate zone in Europe [10, 11] is characterized by low genetic structure and by partial spatial overlap of three mitochondrial clades (defined previously by Michaux *et al*. [12–13]). Such lack of obvious structure is observed in highly mobile species (e.g. wolf, [14]) and is explained by extensive historical or current dispersal and gene flow. However, it remains unknown what are the mechanisms underlying the sympatric occurrence of mitochondrial clades that diverged approximately 0.4–0.6 Ma [12] in a species with limited dispersal capability.

Phylogeography makes use of molecular and geographical data to understand history of a species but often neglects other factors and biological processes that can shape the phylogenetic pattern observed in the contemporary populations of different species [15, 16, 17]. Mitochondrial DNA (mtDNA) − due to simple, uniparental inheritance pattern, lack of recombination, and higher rates of nucleotide substitution in comparison to nuclear DNA − has often been used to examine postglacial colonization and to infer the impact of past barrier [18–19]. Given the fact that mitochondrial-encoded genes are involved in basic metabolic functions (cellular respiration), it was assumed for decades that mitochondrial genome evolved in a manner consistent with the neutral equilibrium model of molecular evolution [20], and adaptive mutations, which would spread through positive selection are very rare [21].

It has been suggested that genes encoded in mtDNA are involved in physiological adaptation to different thermal conditions [22–24]. So as shown in the studies by McDevitt *et al*. [25] on weasels, Silva *et al*. [26] on European anchovy, and Tarnowska *et al*. [17] on bank vole, the distribution and range of different mtDNA lineages can be related to adaptation of certain genetic populations to different climatic and environmental conditions.

Despite the common occurrence of the yellow-necked mouse *Apodemus flavicollis*, there are no many genetic studies on mice and most of them are limited to the phylogeography of the species [12–13]. Moreover, most of the mitochondrial DNA analyses of yellow-necked mice were conducted on individuals from western and southern Europe with limited number of samples collected in the eastern part of the species range (Michaux *et al*., [12–13, 27]. According to Michaux *et al*. [12–13] two highly divergent lineages are observed, one widely distributed in Europe and the second one comprising mice from Turkey, Near and Middle East. Molecular clock analysis showed that separation between these two allopatric lineages occurred between 2.2 and 2.4 My ago, during one of the first Quaternary climate oscillations [12]. Differentiation of clades within European and Russian samples is much lower and separation time between the ancestral haplotypes of the observed three clades was estimated at 0.4–0.6 My [12]. The first clade comprises individuals from all the Western Palearctic regions including western (France, Belgium), northern (Sweden), and eastern Europe as far as Ukraine as well as the three Mediterranean peninsulas (Spain–Portugal, Italy, and the Balkans). The second one is present in northern Europe (Russia, Estonia, Lithuania, Belarus, and Sweden) and the Balkan region (Greece, Romania, and Western Turkey). The third one occurs in southern Russia, Romania, and the Balkans.

The aims of this study was to recognize the phylogeographic pattern and mtDNA structure of yellow-necked mouse population in north-eastern Poland, to compare it with available phylogeographic data of the species in Eurasia, and to answer a question whether climatic variables and habitat directly determine the distribution and frequency of mitochondrial haplogroups of yellow-necked mice. The results of the study fill in the gap in the phylogeography of the most common European rodent species in central Europe and will help to answer the questions if there are any natural factors, other than isolation in LGM refugia and postglacial migration routes, which can have impact on the genetic diversity and spatial structure of the species.

## Material and methods

### Study area

**Fig 1:**
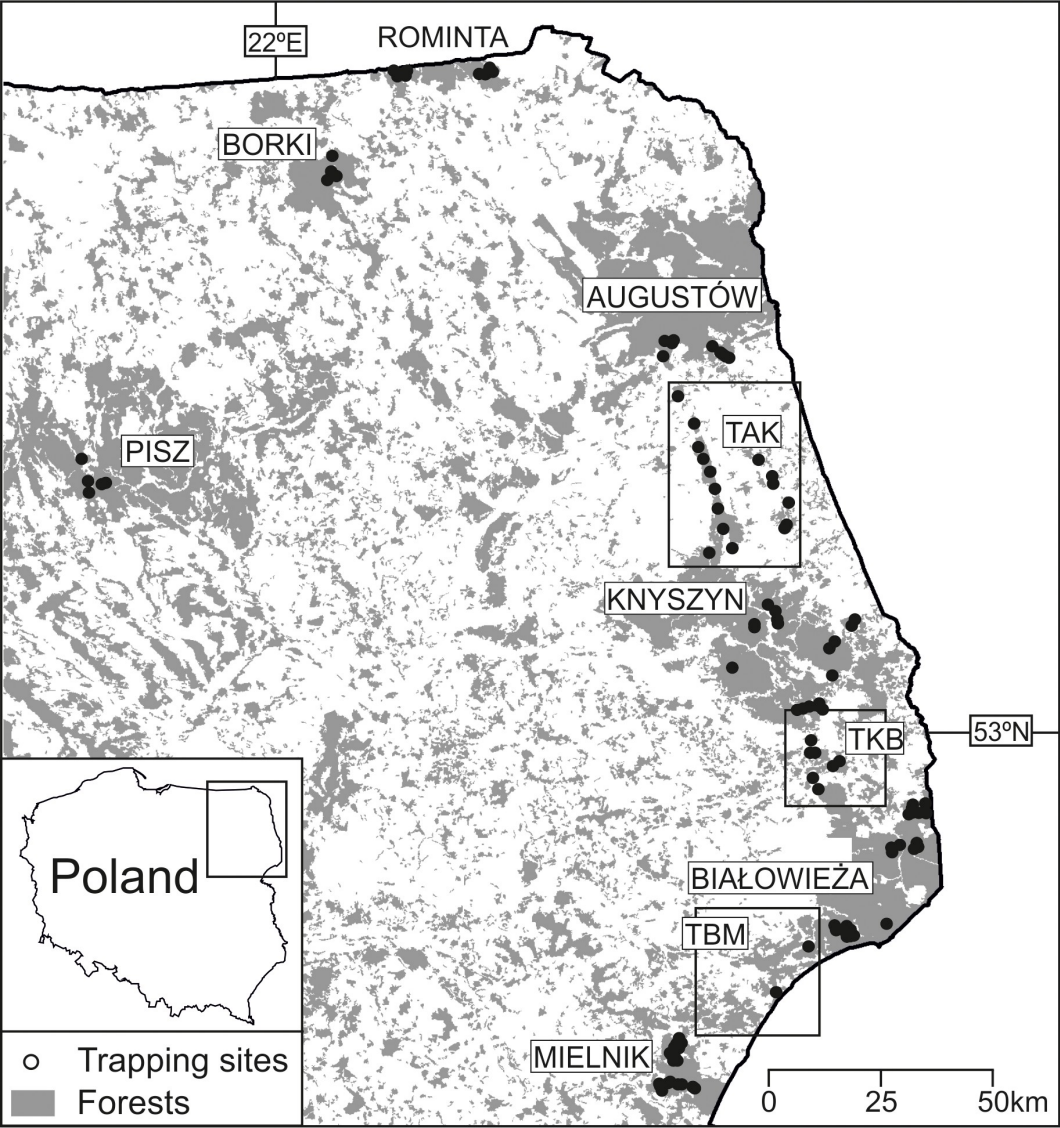
The study area and trapping sites for the yellow-necked mouse *Apodemus flavicollis* mtDNA analyses in NE Poland. Augustów (AUG), Białowieża (BIAL), Borki (BOR), Knyszyn (KNYSZ), Mielnik (MIEL), Pisz (PIS), and Rominta (ROM) forests as well as three transects (T) among four of them: Augustów−Knyszyn (TAK), Knyszyn−Białowieża (TKB), and Białowieża−Mielnik (TBM).

The study was conducted in 7 large woodlands in the lowlands of northeastern Poland (Fig 1): Augustów (AUG), Białowieża (BIAL), Borki (BOR), Knyszyn (KNYSZ), Mielnik (MIEL), Pisz (PISZ) and Rominta (ROM) Forests as well as along three transects (T) consisting of small, fragmented woodlots among four of the above–mentioned forests: Augustów–Knyszyn (TAK), Knyszyn–Białowieża (TKB), and Białowieża–Mielnik (TBM).

The landscape of the study area has been shaped mainly by the Riss, 310 000 to 130 000 yBP, and the Würm, 70 000 to 10 000 yBP, glaciations. It is a plain (altitude between 25 and 312 m a.s.l.), with some belts of frontal and moraine hills and numerous postglacial lakes. Forest cover (29.9%) is slightly higher than the mean for the country (28.7%) [28]. The climate is transitional between continental and Atlantic types with mean January temperature –4°C and mean July temperature 17–18°C, annual precipitation 550–700 mm, snow cover lasting 76–90 days, and the growing season duration of 180–200 days [29].

The study area (53°56'– 54°36' N, 21°04' –23°94' E) spanned over a maximum distance of 230 km in the S–N and 180 km in the E–W direction. Majority of the sampling sites were located in managed forests, several in the national parks in BIAL and AUG forests and in small nature reserves in all forests. The woodlands differed in size (from 126 to 1600 km^2^), productivity, and mean age of tree stands. The most common tree species were Scots pine (*Pinus silvestris*), Norway spruce (*Picea abies*), common oak (*Quercus robur*), silver birch (*Betula pendula*), white birch (*B*. *pubescens*), and common alder (*Alnus glutinosa*). The Białowieża Forest (BIAL) is the oldest and the best preserved woodland in the north-eastern Poland [30]. Woodlots along the transects are smaller and consist of significantly younger, fragmented and often isolated tree stands.

### Trapping of mice

Yellow-necked mice were live-trapped in the seven woodlands (PIS, BOR, ROM, AUG, KNYSZ, BIAL, MIEL) from mid June until early September in 2004–2006 and in four woodlands (AUG, KNYSZ, BIAL, MIEL) and along three transects (TAK, TKB, TBM) from early July to late September in 2007–2008. Trapping and sampling protocols are described in details in Czarnomska *et al*. [31]. In total, within a period of 2004–2008, 820 individuals of yellow-necked mouse were captured at 176 trapping sites (Fig 1). Tissue samples (by ear or tail clipping) were taken from captured mice for DNA analyses. All capture procedures were in accordance with Polish law and were accepted by the Local Ethical Commission in Białystok (permissions nos 07/2004, 15/2006, 65/2007). Trapping site locations were approved by the administrators of the area.

### Laboratory analysis

Tissue samples were stored in tubes with alcohol in –20°C upon DNA extraction. Genomic DNA was extracted using DNeasy Blood & Tissue Kit (QIAGEN) according to the manufacturer’s protocol. Quantity and quality control was performed using spectrophotometer NanoDrop ND–1000. A cytochrome b gene fragment (247 bp) of mitochondrial DNA (mtDNA) was amplified using primers designed to discriminate three sympatric species of *Apodemus*: wood mouse *A*. *sylvaticus*, striped field mouse *A*. *agrarius* and yellow–necked mouse [32]. Amplification reaction was carried out in 25 μL volumes including 2 μL of each 5pM primer, 2.5 μL of 2mM dNTP, 2.5 μL of 10x reaction buffer, 12.1 μL of purified water and 0.4 μL of 5U Maxima Hot Start Taq DNA Polymerase (Fermentas) and 2 μL of DNA. PCR reaction was performed in 33 cycles (20 s at 94°C, 30 s at 58°C and 1 min 30 s at 68°C) with initial step of denaturation for 15 min at 94°C and final elongation for 10 min at 68°C. PCR reactions were carried out using a DNA Engine Dyad Peltier Thermal Cycler (BIO RAD). PCR product was evaluated on 1% agarose gel and only good quality samples were purified using Clean Up kit (A&A Biotechnology, Gdańsk, Poland) and directly sequenced using Big Dye Terminator Cycle Sequencing Kit (Applied Biosystem). The PCR product was purified using ExTerminator kit (A&A Biotechnology) and sequences of mtDNA were analysed using DNA Sequencing Analysis ver. 3.0. implemented on ABI PRISM 3100 Genetic Analyzer (Applied Biosystems). A final data set consisted of 353 samples with mtDNA haplotype identified. Detailed information on the number of analysed samples from each forest, transect, and year is presented in S1 Table.

### Statistical analysis of genetic data

Sequences of analysed mtDNA fragment were aligned and visually inspected in program BioEdit ver.7.0.9.1 [33]. Obtained sequences representing different haplotypes were deposited in the GenBank database (Accession numbers MK840479—MK840502). To evaluate whether the number of analysed samples is representative for study area and to estimate the total number of haplotypes, rarefaction curve was constructed by plotting the cumulative number of haplotypes found with increasing sample size. The total number of haplotypes was estimated as the asymptote of this curve [34]. As the sampling order affects the shape of the curve, the data set was randomized 1000 times using GIMLET [35] and 1000 rarefaction curves were generated using R [36] and a script file produced by GIMLET. The asymptote (a) for each curve was calculated from the equation: y = ax/(b+x), where y is the cumulative number of haplotypes, x is the number of sampled individuals, and b is the rate of decline in the slope of the curve [34]. The number of haplotypes was estimated as the mean value of the asymptote a for all iterations.

Phylogenetic analyses were done in two steps: first using only haplotypes detected in this study and second combining them with 370 sequences known from other studies ([12–13, 27, 32, 37–45], Essbauer *et al*., unpubl.; Guenther *et al*., unpubl.; Splettstoesser *et al*., unpubl.) available at NCBI GenBank website. Sequences deposited in NCBI database differed in length, therefore for a purpose of phylogenetic tree and haplotype network construction only a fragment of 247 bp used in this project was selected. It resulted in 52 additional sequences of *A*. *flavicollis* from Europe and Near East (S2 Table). In case when the fragment of 247 bp of sequences from different geographical locations were identical, such haplotype was used only once but that information was used for interpretation of results (S2 Table). Despite the short length of the analysed fragment it has 65 variable nucleotides (corresponding to 25% of the sequence) among all used sequences and 55 variable sites among European samples.

To determine the model of nucleotide substitution that best fitted our data, FINDMODEL software was used (http://www.hiv.lanl.gov/content/sequence/findmodel/findmodel.html). The program implements the algorithm developed for MODELTEST [46] using scores for likelihood of trees generated under 28 compared models. Software MEGA ver.5 [47] was used to construct phylogenetic trees using Maximum Parsimony approach, Maximum Likelihood method (model HKY), Neighbour–Joining method using Tamura Nei model (TrN) with a shape parameter of the gamma distribution, the most similar to best fit model HKY from available models. Moreover, median–joining network was constructed with program Network [48]. For better visualization of relationship among distinct haplotypes only fact of occurrence, disregarding their frequencies, was considered for the construction of the network.

To infer population genetic structure based on mtDNA haplotype frequency, we applied the spatial analysis of molecular variance implemented in the SAMOVA software [49]. This approach defines groups of populations that are geographically homogenous and maximally differentiated from each other. Sampling localities were arranged in 10 regions (seven forests and three transects). The method is based on a simulated annealing procedure that aims to maximize the proportion of total genetic variance due to differences between groups of populations. This approach requires the a priori definition of the number (K) of groups. SAMOVA was ran with K ranging from 2 to 9. An analysis was performed twice to check for the consistency of results between runs. In each run, 100 simulated annealing processes were performed. The recognition of the most probable number of groups was based on the pattern of changes in values of Φ–statistics parameters with K. Additionally, to control for bias due to overrepresentation of samples from southern part of BIAL collected in 2007, SAMOVA was run twice on randomly chosen reduced subset of samples from this location for comparison.

Genetic variability parameters for mtDNA sequences (the number of haplotypes and polymorphic sites, haplotype and nucleotide diversity within each defined subpopulations by SAMOVA) were estimated using DnaSP ver. 5 [50]. While haplotype diversity (which reflects a probability that two haplotypes randomly chosen from a sample are different) is based on haplotype frequency alone, nucleotide diversity also takes into account the amount of divergence among the haplotypes found in a sample. Pair-wise genetic differentiation among subpopulations inferred in SAMOVA was calculated in Arlequin ver. 3.11 [51].

### Association of genetic variation with environmental factors

Each mtDNA haplotype was assigned to one of three confirmed haplogroups. For each study site (the forest or the transect) and trapping year, which had at least 5 assigned haplotypes, we calculated the proportion of haplotypes from each of the haplogroups as well as mean temperatures of January and July calculated for the year of sampling and the percentage cover of deciduous-mixed forests (preferred habitat of yellow-necked mouse). The cover of deciduous-mix ed forest was measured in 1-km buffer zone around trapping siteson the basis of the Corine Land Cover 2006 database (European Environment Agency, 2013) using the ArcView GIS software by ESRI (version 9.3.1).The mean temperatures of January and July were calculated by averaging 4 measurements per each month of land surface temperature with high spatial (grid 1×1 km) resolution (LST–MODIS data set; grid 1x1 km, [52], NASA LP DAAC 2016).

To verify which of the considered environmental factors affected the probability of haplotype assignment to a given haplogroup, we fitted generalized linear mixed models (GLMMs) for binomial data using the “glmer” function implemented in the lme4 package [53]. We set three global models differing in binomial (response) variables. In the first global model, “1” was attributed to the haplotypes assigned to Haplogroup 1 and “0” to haplotypes from Haplogroups 2 and 3. In the second global model, “1” was assigned to haplotypes from Haplogroup 2 and “0” to haplotypes from Haplogroups 1 and 3. In the third global model, “1” meant haplotypes from Haplogroup 3 and “0” haplotypes from Haplogroups 1 and 2. As the collinearity between explanatory variables was negligible (|*R*|< 0.35), all variables (i.e. mean temperature of January, mean temperature of July and percentage cover of deciduous-mixed forests) were included in the global model. We applied GLMM with observation-level random effect instead of generalized linear model (GLM) because results of the latter indicated an excess of variation in the data which caused considerable overdispersion.We applied an information theoretic approach with a second-order correction for small sample size (AIC_*c*_ [54]) implemented in R package MuMIn [55] to select the submodel the variables of which best explained the observed haplotype assignment to a given haplogroup. Statistical analyses were performed in R [36].

On the pan-European scale, we plotted the distribution of different mtDNA haplogroups of the yellow-necked mouse against the mean January and mean July temperatures (°C) obtained from the WorldClime database (resolution ~ 1 km^2^ [56]).

## Results

### Mitochondrial DNA variability and phylogeny

A total of 24 mtDNA haplotypes (H1–H24), defined at 22 polymorphic sites (in which eight were parsimony informative), were recorded in 353 analysed samples, with only six of these reported previously in other studies (S2 Table). The remaining haplotypes were newly identified. Estimated haplotype diversity was 0.88 (SD 0.009) and nucleotide diversity was 0.01 (SD 0.0003). Only six haplotypes were represented by more than 5% of studied individuals each, and the most common of them (H7) constituted 23.8% of the entire sample. Four of the most abundant haplotypes were known from previous studies. Five haplotypes were found only once. The expected number of haplotypes, estimated by rarefaction curve (S1 Fig), was 26 (mean: 26.4, SE 1.6, median 26.3, range 22–32). Therefore, the analysed sample of mice is representative for the studied area, as 92% of expected haplotypes were detected.

**Fig 2:**
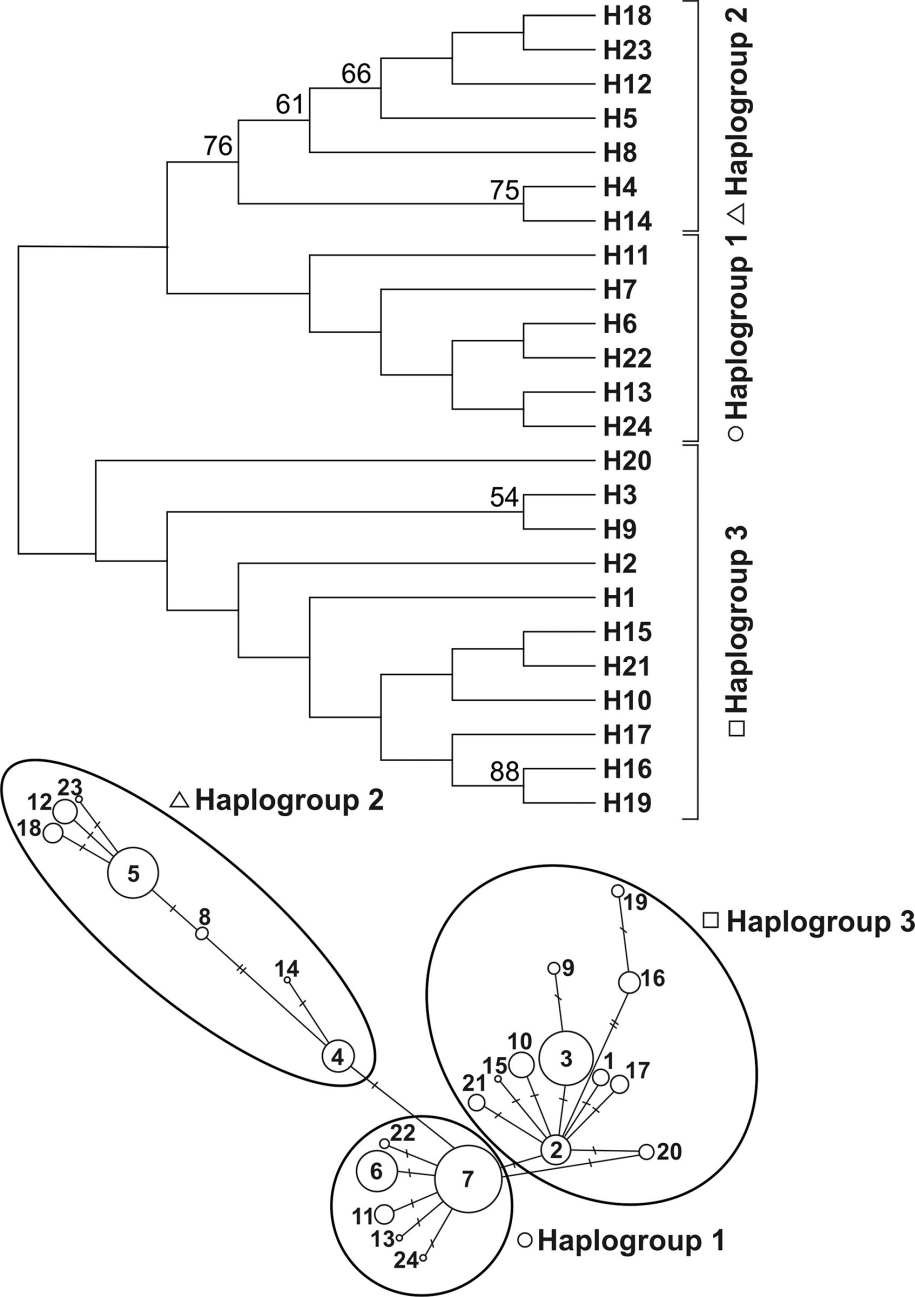
Upper panel: Neighbour–joining tree phylogenetic tree of cyt b fragment of mtDNA haplotypes of yellow-necked mouse found in this study. Lower panel: Median joining network constructed in program NETWORK. Dashes denote number of mutation steps among haplotypes.

**Fig 3:**
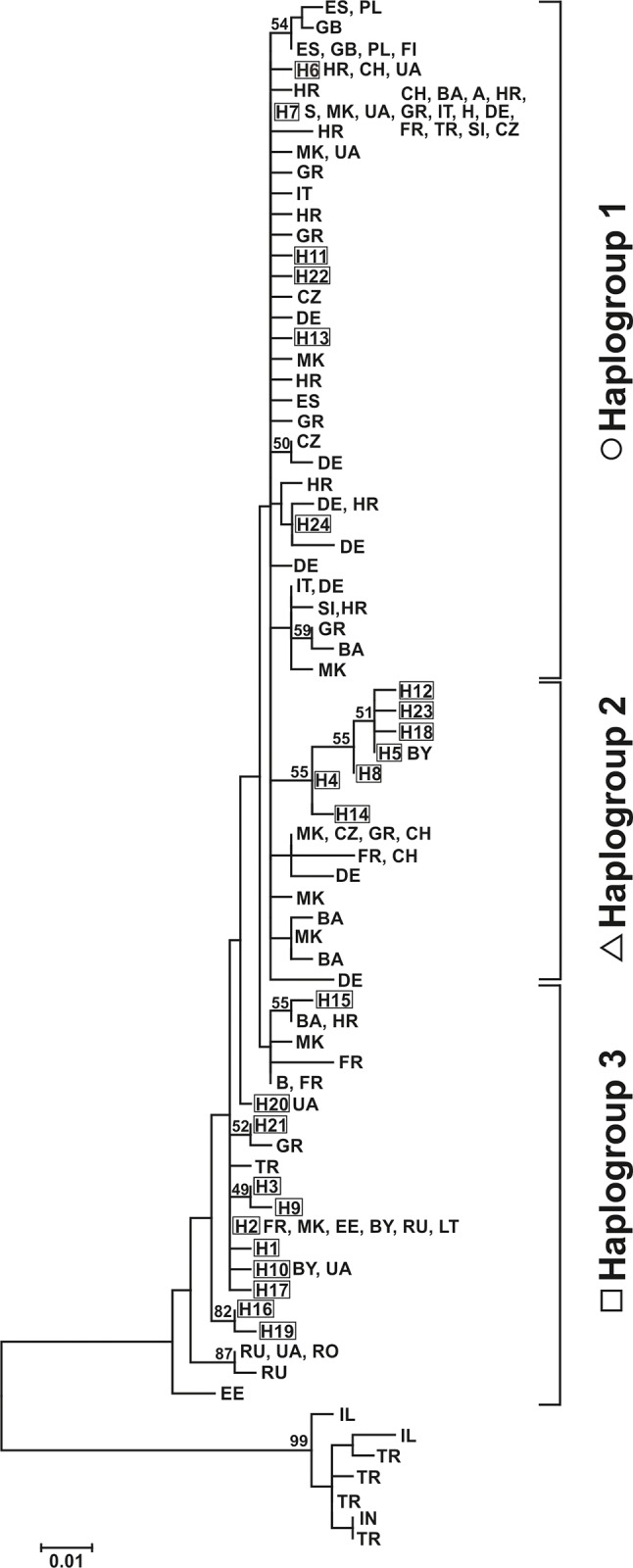
Maximum likelihood tree of cyt *b* fragment of mtDNA haplotypes of yellow–necked mouse found in NE Poland (H1 − H24) and recorded in other studies. Bootstrap values shown if greater than 50%. Sequences from GenBank are coded according to the country location. List of data and sources in S2 Table.

Phylogenetic relationship among 24 haplotypes found in NE Poland was expressed in the form of a Neighbour Joining phylogenetic tree and median-joining network (Fig 2). There was a high consistency in topology of the tree and the median joining network, generally supporting the presence of three haplogroups (Fig 2). Nevertheless, phylogenetic distinction of haplotypes that formed Haplogroup 2 was detected with the highest statistical support and formed a distinct group also when compared with published data from Europe (Fig 3). Higher bootstrap values were obtained only for two haplotypes (H16, H19) belonging to Haplogroup 3 (Fig 2). Evolutionary divergence between the analysed sequences was estimated with Kimura 2-parametric model and was ranging from 0.004 to 0.03 (mean 0.015).

The median-joining network (Fig 2) has a star–like shape with most of haplotypes differentiated by single mutation step with the exception of H5, H8, H12, H18, H23 (belonging to Haplogroup 2) and H16, H19 (Haplogroup 3). Greater level of differentiation of the above mentioned haplotypes was confirmed also by the phylogenetic tree.

**Fig 4:**
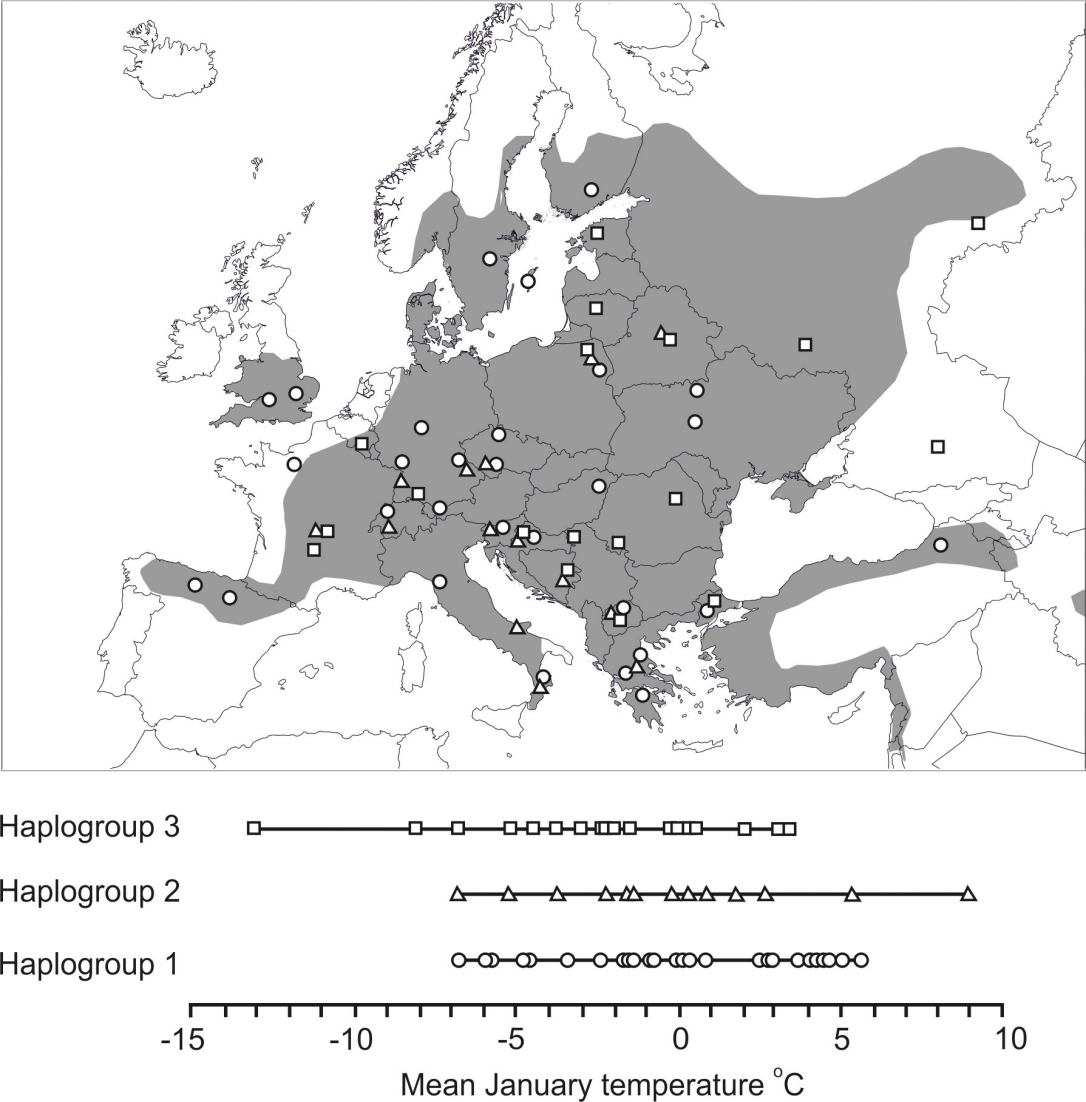
Geographic distribution of cyt *b* haplotypes of yellow-necked mice based on sequences derived from this study and the analoque cyt *b* fragment from the published data (sources in S2 Table). Lower panel: Mean January temperature (°C) in each of the sampling points, where the individuals belonging to haplogroups 1–3 were found. Shapes of symbols indicate assignment to haplogroups based on Maximum Likelihood phylogenetic tree: circles–Haplogroup 1, triangles–Haplogroup 2, squares Haplogroup 3. In dark grey: the range of the yellownecked mouse in Europe (from: www.iucnredlist.org).

Haplotypes derived from this project were assigned to three mtDNA subclades described by Michaux *et al*. [12]. Haplotypes from Haplogroup 1 clustered with haplotypes comprising “western” clade (1a –Michaux *et al*. [12]), Haplogroup 2 had intermediate position with Belarusian sample (1b –Michaux *et al*. [12]) being identical to H5 recorded in north–eastern Poland, and Haplogroup 3 clustered with the “south–eastern” clade (1c –Michaux *et al*. [12]). Comparison of our results with the published data showed that all three mtDNA haplogroups partly overlap in their spatial distribution in Europe (Fig 4). Haplogroup 3 occurs further to north-east (cold and dry continental climate) than other two groups. Haplogroup 1 is the sole group occurring in the western and north-western parts (mild and humid) of the species' range, and Haplogroup 2 has the smallest and the most central range of the three groups (Fig 4).

### Spatial genetic structure based on frequencies of mtDNA haplotypes

Spatial distribution of identified haplotypes (S3 Table) showed that genetic differentiation among studied regions in the north-eastern Poland is not reflected by geographic fixation of the detected haplotypes. Nevertheless, haplotypes identified in northern part of study area (ROM, BOR, PIS) clearly showed restricted spatial distribution with H21, H20, H19 found only in ROM and H16 occurred in ROM, PIS, TAK, KNYSZ, while haplotypes H5, H8, H12, H18, and H23 were more common in southern regions. Majority of haplotypes occurred over the whole studied area, but they considerably differed in frequency in various forests (S3 Table).

**Fig 5:**
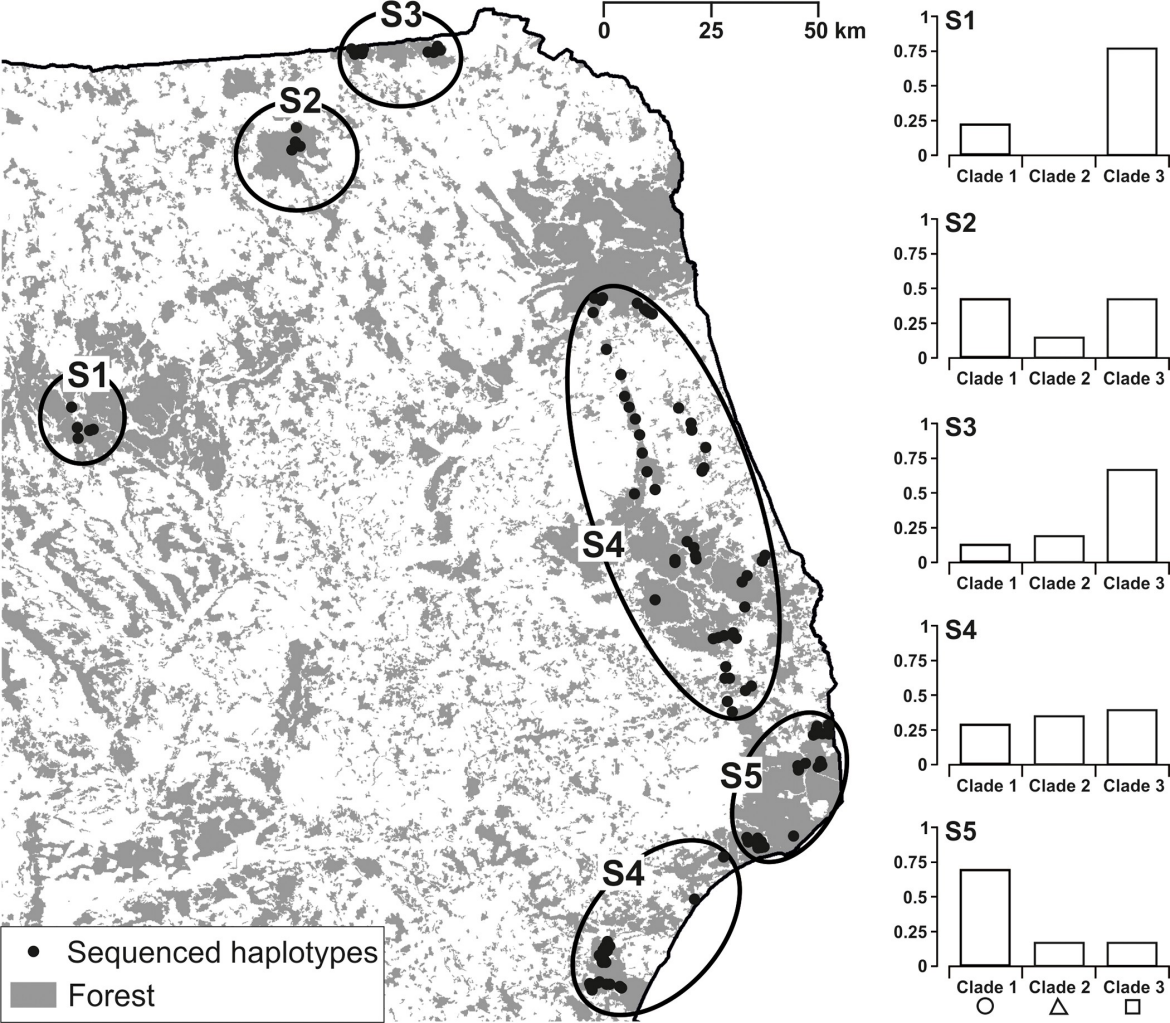
Five subpopulations (S1–S5) of yellow-necked mouse in north-eastern Poland, based on mtDNA haplotype frequency, delimited by SAMOVA (left panel). Proportions of mtDNA haplogroups (clades) in each of the defined subpopulation (right panel). Black points refer to trapping sites.

SAMOVA was used to assess the spatial structure taking into account geographic location and mitochondrial haplotype frequency. The results of SAMOVA indicated significant population genetic structure for each assumed number of groups (2–9). The primal division (for two groups) corresponded to separation of PIS from the remaining regions. The highest increase in Φ_CT_ value occurred between K = 3 and K = 5 and all parameters of the Φ–statistics stabilized at K = 5 (S2 Fig). Thus, 5 groups were assumed as the most parsimonious clustering that maximized variation among groups. Individuals from PIS, BOR, ROM, and BIAL formed four distinct subpopulations (Fig 5). Mice from AUG, KNYSZ, MIEL and the three studied transects formed geographically disjunct subpopulation S4 (Fig 5). This group consisting of individuals from three forests and three transects was stable from K = 3 to K = 5 and with increasing K further subdivision was observed. Distinction of BIAL subpopulation (S5) was supported by analyses of both the full data set and the randomly reduced set to control for the possible bias due to large number of individuals captured there in 2007. In case of five spatial groups the variation within local populations accounts for 87.2% of the total variation, but variation among populations within defined groups accounts for only 4.14% of the total. Slightly bigger variation was observed among groups (8.66% of the total variation).

Although there was no clear pattern in spatial distribution of single haplotypes, such pattern was visible at the level of haplogroups. Haplotypes belonging to Haplogroup 1 dominated in the southeastern part of the study area (up to 69% in BIAL Forest, S5). Haplotypes of Haplogroup 2 (H4, H5, H8, H12, H14, H18) made up 27% of all identified haplotypes in the whole study area and were most common in S4–34% (Fig 5, S3 Table). Haplotypes that formed Haplogroup 3 were found in 37% of all mice and highly prevailed in the northwestern region (78% in PIS, S1) with substantially reduced occurrence in southeastern region (16% in BIAL, S5).

Pairwise Ф_ST_ values calculated among five subpopulations (S1–S5) were moderate but highly significant (Table 1). Defined subpopulations showed substantial differences in haplotype diversity (Table 2). The highest diversity was found in subpopulations S3 (ROM) and S4, the most numerous group. The number of haplotypes found in subpopulations was highly correlated with the number of analysed samples (*r* = 0.97, *p* = 0.005) but haplotype diversity did not show such correlation (*r* = 0.15, *p* = 0.806).

**Table 1:** Pair-wise genetic differentation coefficients (Φ_ST_) among five subpopulations of yellow-necked mice in NE Poland, defined by SAMOVA (see map in Fig 5). All values were statistically significant at *p* < 0.005 after Bonferroni correction (*k* = 10) except that marked by asterisk (*).

Subpopulation	S1	S2	S3	S4
S2	0.195	–		
S3	0.133	0.102	–	
S4	0.121	0.121	0.098	–
S5	0.285	0.112*	0.175	0.106

* *p* = 0.009

**Table 2:** Mitochondrial DNA variation parameters calculated in subpopulations identified by SAMOVA. See Fig 5 for spatial distribution of subpopulations S1–S5.

Parameter	S1	S2	S3	S4	S5
Sample size	9	14	30	214	86
Number of haplotypes	5	6	8	14	9
Number of polymorphic sites	6	9	10	15	9
Haplotype diversity (SD)	0.833 (0.098)	0.780 (0.081)	0.876 (0.023)	0.875 (0.009)	0.663 (0.049)
Nucleotide diversity (SD)	0.009 (0.001)	0.009 (0.002)	0.012 (0.001)	0.012 (0.000)	0.006 (0.001)

### Genetic structure related to climate

**Fig 6:**
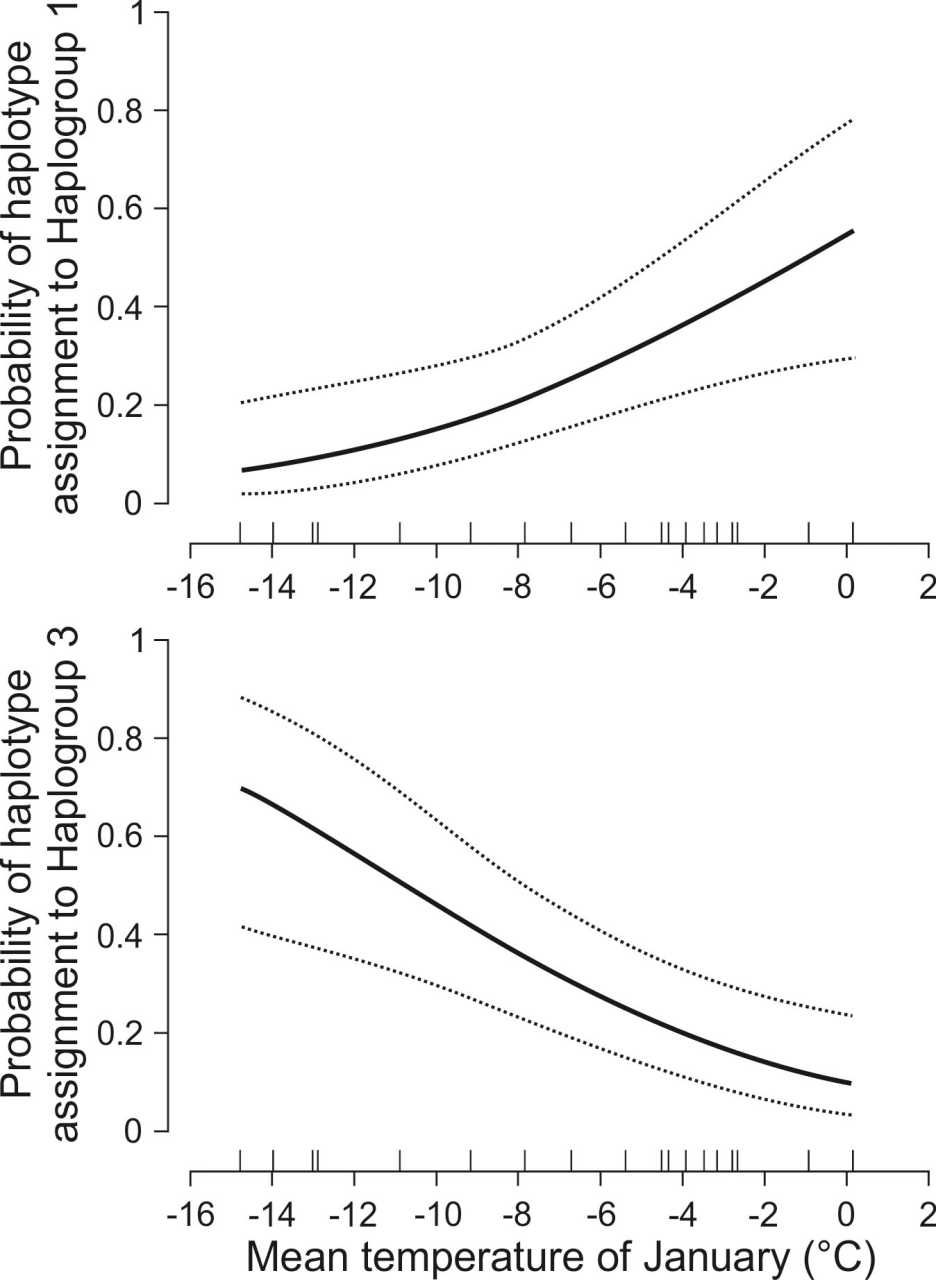
Relationships between mean temperature of January in the year of trapping in 10 studied region in NE Poland and probability of yellow-necked mouse assignment to Haplogroup 1 (upper panel) and Haplogroup 3 (lower panel) − results of the most parsimonious generalized linear mixed models (GLMMs) presented in Table 3.

The observed variation in climatic and habitat conditions across the study area (S4 Table) suggested that environmental factors might have been also of relevance to explain the haplotype assignment. AIC ranking indicated that among considered combinations of submodels (GLMMs) for Haplogroups 1 and 3, the models with mean temperature of January as the only explanatory variable was both the simplest and had the lowest AIC_*c*_ scores (Table 3). In the case of haplogroup 2, there was no indication of environmental effects on the probability of haplotype assignment: the top AIC rank was attributed to the intercept model. Therefore, for Haplogroups 1 and 3, we selected models with mean temperature of January (calculated for the year that preceded sample collection) as single best models. The GLMMs indicated significant positive effect of increasing mean temperature of January on the probability of haplotype assignment to Haplogroup 1 (slope = 0.19 ± 0.07, *z* = 2.74, *P* = 0.006) and negative effect on the probability of haplotype assignment to Haplogroup 3 (slope = -0.21 ± 0.07, *z* = -3.15, *P* = 0.002). With increasing mean temperature of January from -14.8°C to 0.1°C the probability of haplotype assignment to Haplogroup 1 increased from 7 to 56%, while the probability of haplotype assignment to Haplogroup 3 decreased from 70% to 10% (Fig 6). These relations were not statistically significant when mean temperature of January calculated for all years of trapping was used for the analyses.

**Table 3:** Model selection (based on the AICc criteria) for the considered GLMMs which aimed at assessing the effect of mean temperature of January (T_JAN_), mean temperature of July (T_JUL_) and percentage cover of deciduous-mixed forests (DMF) on the probability of haplotype assignment to Haplogroups 1, 2 and 3.

Model	K	AIC_*c*_	ΔAIC_*c*_	ω_*i*_
Haplogroup 1				
T_JAN_	3	160.8	0.00	0.48
T_JAN_+T_JUL_	4	163.0	2.19	0.16
T_JAN_ +DMF	4	163.6	2.76	0.12
Intercept	2	164.4	3.60	0.08
T_JUL_	3	165.0	4.20	0.06
T_JAN_ +T_JUL_ +DMF	5	165.4.2	4.62	0.05
DMF	3	167.0	6.19	0.02
T_JUL_ +DMF	4	167.0	6.20	0.02
Haplogroup 2				
Intercept	2	164.0	0.00	0.37
DMF	3	164.4	0.45	0.30
T_JAN_	3	166.8	2.81	0.09
T_JUL_	3	166.8	2.86	0.09
T_JAN_ +DMF	4	167.7	3.72	0.06
T_JUL_ + DMF	4	167.7	3.72	0.06
T_JAN_ + T_JUL_	4	170.1	6.15	0.02
T_JAN_ + T_JUL_ + DMF	5	171.5	7.49	0.01
Haplogroup 3				
T_JAN_	3	164.5	0.00	0.54
T_JAN_+T_JUL_	4	166.3	1.78	0.22
T_JAN_ +DMF	4	167.8	3.29	0.10
Intercept	2	169.7	5.19	0.04
T_JUL_	3	169.8	5.28	0.04
T_JAN_+T_JUL_ +DMF	5	170.2	5.67	0.03
DMF	3	172.5	8.04	0.01
T_JUL_ +DMF	4	173.0	8.53	0.01

*K*, the number of estimated parameters; AIC_*c*_, Akaike’s Information Criterion with a second-order correction for small sample sizes; ΔAIC_*c*_, difference in AIC_*c*_ between the specific model and the most parsimonious model;ω_*i*_−Akaike weights.

On the pan-European scale, there were no statistical significant differences (Kruskal-Wallis test) among either mean January or mean July temperatures in study sites, where different mtDNA haplogroups were found (January: H = 3.744, df = 2, N = 61, p = 0.1538, and July: H = 0.891, df = 2, N = 61, p = 0.6404). However, the study sites, where haplogroup 3 occurred, indeed spanned over colder January temperatures <-13.1oC, 3.4oC> than those of the two other haplogroups (Fig 4, lower panel).

## Discussion

### Genetic variation and differentiation among local populations

Overall, the yellow-necked mouse population inhabiting north–eastern Poland was characterized by moderate level of mitochondrial DNA diversity. Mean haplotype diversity was moderately high, whereas nucleotide diversity was low, indicating the presence of a large number of closely related haplotypes. Number of detected haplotypes, approximately one per 15 analysed samples, was low when compared to other studies [12, 44, 57]. Such findings correspond well to the hypothesis of gradual decrease of genetic variation with the increasing geographic distance from the presumed LGM refugium in one of the South Mediterranean peninsulas [2, 18]. Saturation of rarefaction curve at the number of 26 haplotypes indicates that most of the variants within the analysed fragment of cytochrome *b* were detected.

Haplotypes found in north–eastern Poland were not geographically fixed but substantially differed in frequency among the studied forests. Inferences in program SAMOVA defined five genetic groups with subpopulation inhabiting Pisz Forest being genetically most distinct from the remaining samples. Such distinction of Pisz Forest was supported furthermore by genetic differentiation indices (Φ_ST_ and F_ST_) and might be related to high habitat fragmentation observed in that forest [31, 58–59]. SAMOVA results have shown that high percent of a total observed variation accounts for the variation within local populations and very low amount of variation was observed among populations within defined spatial groups. This suggests that there is overall high gene flow among local populations and between defined groups. Although SAMOVA’s authors noted that the program performance is less reliable,when working with populations slightly differentiated and influenced by isolation of distance [49], it does not seem to cause a spurious substructuring in the analysed data. Thus, defining five maximally homogenous groups seems reasonable. Subpopulations S4 and S3 exhibited the highest mtDNA variability. Although it is justifiable that subpopulation S4 that spanned over large distance and included over half of analyses samples is characterized by great genetic diversity, the variation observed in Rominta Forest (subpopulation S3) is difficult to explain. Located at the northernmost part of study area, it might be connected to a larger, transborder population, not covered by sampling, that substantially differed in genetic composition. On the contrary, Białowieża subpopulation (S5), despite relatively large sample size, was characterized by the lowest mtDNA variation and a large representation of H7, haplotype most common in the whole European population of yellow–necked mouse (see S2 Table).

### Phylogeography of yellow-necked mouse

In phylogenetic comparison we refer to topology defined by Michaux *et al*. [12, 13] as their phylogeographic analyses remain the most extensive study on yellow–necked mouse conducted in Europe. Since then several areas were covered by additional sampling, e.g. Ukraine [57], Balkans [44–45] but those projects were focused on local populations and none of them aimed at comprehensive update of species phylogeography in Europe. According to Michaux *et al*. [12–13], two main lineages of yellow–necked mice are observed, one widely distributed in continental Europe and the second one comprising mice from Turkey, Near and Middle East. High differentiation among the two lineages (level comparable with subspecies status) had strong statistical support in all phylogenetical analyses [12]. Differentiation of clades within European and Russian samples was much lower and intraspecific structure of these continental clades developed later, during middle to late Quaternary. Michaux et al. [12] used the term subclades to describe *A*. *flavicollis* phylogeography within European continent. To avoid confusion, we are using the term haplogroups, when discussing European samples and the term lineage when referring to populations from Turkey and Near East regions.

Comparison of sequences derived from this project and 52 sequences from other studies in Europe resulted in structure highly concordant with that suggested by Michaux *et al*. [12, 13]. Although most of internal branching did not receive bootstrap support, three haplogroups within Europe were detected. There are, however, small differences in topology and misassignments in regards to formerly presented phylogenetical trees.

Twenty-four mitochondrial cyt *b* gene haplotypes detected in NE Poland belonged to three European haplogroups. Haplotypes that clustered into Haplogroup 2 had the highest statistical support and formed a distinct group also when compared with published data from Europe. Distribution of Haplogroup 2 haplotypes was not restricted to any particular region of north–eastern Poland. Moreover, analogue sequence to haplotype H5 (the most numerous among Haplogroup 2) was found in Belarus [12]. It might indicate that Haplogroup 2 is frequent in central-eastern Europe and its presence remained underestimated due to scarce data available from that region. Although the majority of identified haplotypes (18 out of 24) have not been recorded by other researchers, four of the most abundant haplotypes from our study area have sequence equivalents widely distributed in Europe, especially H7 which was found from France to Turkey (see S2 Table). On the other hand H3, recorded in 58 analysed individuals (16.4%) and found in most of the surveyed forests of north–eastern Poland, was never recorded outside this area. These findings, however, might be affected by a limited number of samples from eastern Europe used in previous studies [12, 13].

Low level of differentiation between mtDNA haplotypes recorded in yellow-necked mouse in north–eastern Poland resulted in star–like shape of the median–joining networks, which is in high concordance with published data [12, 13]: the lineages of yellow-necked mice (Europe versus Near East and Turkey) were separated by 46 mutational steps, whereas European haplogroups by five and nine mutational steps, respectively. Comparison of 247 bp mitochondrial gene fragment showed the consistency of this structure pattern although with lower resolution (22 mutational steps detected between two lineages of mice).

The network shape showed that the two haplogroups (1 and 3) stem from H7 and H2, respectively. H7 occurs widely in Europe, whereas H2 has mostly been recorded in eastern Europe (see S2 Table).

### Adaptive mtDNA genetic variation

It was commonly believed that during the glacial maxima, the occurrence of temperate and boreal species was restricted to the Mediterranean refuge areas [2, 60]. Based on yellow–necked mouse phylogeography it was assumed that the Balkan region, where all three postulated European haplogroups coexist, was the source population from which the whole European range of the species was established [12, 13]. Application of climate niche modeling of temperate forest range in LGM by Fløjgaard *et al*. [61] supported the phylogeographic results, which indicated a survival of *Apodemus sp*. in Mediterranean refugia, southern France and Black Sea area, but additionally suggested the southern parts of Russian plains as an area with suitable climatic conditions for temperate species during the glacial maximum. Results of our study also suggest the additional, eastern source of the contemporary European population. It might indicate better adaptation to harsh winter conditions in mice which exhibit cytochrome *b* gene variants of Haplogroup 3, presumably of eastern origin.

Mean January temperature in the field work period in NE Poland correlated with the frequency of mtDNA haplotypes belonging to Haplogroup 3 (negative correlation) and Haplogroup 1 (positive correlation with temperature). Such a strong regional pattern may reflect a genuine genetic structure of the examined population only, as the pan-European spatial ranges of possibly 'thermophilous' Haplogroups 1 and 2 and more 'cold-resistant' Haplogroup 3 largely overlap (see Fig 4).

The mitochondrial cytochrome *b* gene encodes an integral membrane protein component of the cytochrome *bc1* complex, which catalyzes the redox transfer of electrons from ubiquinone to cytochrome *c* in the mitochondrial electron transport chain. Several investigators have already suggested that functional modifications of enzyme involved in mitochondrial oxidative phosphorylation (OXPHOS), such as cyt *b*, may be involved in physiological adaptation to different thermal environments [22–24]. Most of them argued that–in humans–it reflects historical adaptation to different climatic regimes during human expansion out of Africa [22–23, 62] or–in mammals–recolonization from different LGM refugia [17, 25]. For instance, in weasels *Mustela nivalis* it was found that climate significantly affect the current distribution of the Carpathian lineage [25] and that individuals from this lineage might have selective advantage over animals from the other lineage in surviving harsh winter condition in Białowieża Forest. Also, the study of Silva et al. [26] showed that water temperature shaped the contemporary distribution of mitochondrialDNA clade frequencies in the European anchovy.

Importantly, ATP production via OXPHOS complexes critically depends on interaction between mitochondrial and nuclear genomes (mitonuclear interactions), which evidences that the evolutionary trajectories of the two genomes are tightly interlaced [63–64]. Interestingly, the analysis of nuclear DNA (13 microsatellite loci) variation in the studied population of mice [31] resulted in − similar to mtDNA haplogroups − north-south gradient of exchange of the genetic clusters. That spatial pattern of nuclear DNA was best explained by environmental variables, especially January temperature. Mice belonging to microsatellite Cluster 1 (corresponding to mtDNA Haplogroup 3) prevailed in colder regions and in the boreal-type, coniferous forests [31]. Concordant regional-scale patterns of mtDNA and nuclear variation in animals, such as that found in the yellow-necked mice in this study, may be a sign of coadaptive processes in the mitonuclear interaction modyfing physiological performance of animals in cold climates Therfore, it is possible that − in mice population in NE Poland − selection could be acting on nuclear genome, and mtDNA genes might segregate in those patterns as a byproduct of the tight coevolution with nuclear genes.

Although further studies are needed to make firm conclusions, one may suggest that low January temperatures may have impact on contemporary populations of mice by inducing higher survival rates of individuals that belong to mtDNA Haplogroup 3 and microsatellite Cluster 1. It was already found by Wójcik [65] that − in a woodland in E Poland − mice surviving winter were not a random representation of population, and natural selection may be responsible for maintenance of observed polymorphism in transferrin locus. It was suggested that heterozygotes had selective advantage in winter survival due to the transferrin role in resisting microbial infections. Nevertheless, the maintenance of all three mitochondrial clades, despite potentially higher winter survival by individuals from Haplogroup 3, may indicate a dynamic balance, where all haplogroups have some ecological and evolutionary advantages in given environmental conditions. Long-term studies on local populations would further elucidate the role of winter severity on year-to-year variation in mitochondrial and nuclear genetic variation in yellow-necked mice.

### Conclusions

A total of 24 mitochondrial cytochrome *b* haplotypes (including 18 new ones) were identified in NE Poland. They exhibited an overall low level of differentiation and were assigned to three known European haplogroups. The results suggested presence of additional, eastern source of contemporary European population of yellow-necked mouse, apart from the recognized Balkan LGM refugium. The majority of haplotypes occurred over the whole study area but they considerably differed in frequency among regions. SAMOVA defined five genetic groups with subpopulation inhabiting Pisz Forest found to be the most distinct and separated from the remaining samples. Individuals from Borki, Rominta and Białowieża Forests formed three distinct subpopulations. Mice from Augustów, Knyszyn, Mielnik Forests and the three studied transects were clustered in one, yet geographically disjunct, subpopulation. SAMOVA results suggested a high gene flow among local populations and among the defined groups. Mean January temperature was the most relevant environmental predictor of the prevalence of mtDNA haplotypes belonging to Haplogroups 1 and 3. However, to confirm the hypothesis that mice with haplotypes from Haplogroup 3 (presumably of eastern origin) have better adaptation to survive harsh winter conditions, further studies are needed.

## Supporting information

S1 Table Number of samples successfully analysed per geographical region (mtDNA). Numbers of identified mtDNA haplotypes that belong to each defined mtDNA haplogroup.
(DOCX)

S2 Table Yellow–necked mouse cyt b mtDNA sequences available at NCBI GenBank used for comparison with material from this study. (DOCX)

S3 Table Distribution and number of cyt b mtDNA haplotypes detected in samples collected in seven forests and three transects: ROM–Rominta Forest, BOR–Borki, PIS–Pisz, AUG–Augustów, BIAŁ –Białowieża, MIEL–Mielnik, TAK–Augustów-Knyszyn Transect, TKB–Knyszyn-Białowieża, TBM–Białowieża-Mielnik. The numbers of haplogroup of each of the haplotypes are in brackets.
(DOCX)

S4 Table Ecological characteristics of the studied forests and transects and abundance indices of yellow-necked mice. See Table 1 for abbreviations of regions and Fig 2 for their location. Percentage of land use categories based on Corine Landcover 2006 (CLC2006) data in 1-km buffer zone around each trapping site. Mean temperature collected based on four measurements per month of land surface temperature in a spatial grid 1 km × 1 km (MODIS).
(DOCX)

S1 Fig The rarefaction curve presenting a dependence of number of detected haplotypes on a number of analysed samples. (PDF)

S2 Fig Changes in Φ statistics for K = 2 to 9 subpopulations of yellow–necked mice in north-eastern Poland, on the basis of mtDNA and inferred from SAMOVA. Φ_SC_−proportion of the variance among local populations within groups. Φ_ST_−proportion of the variance among local populations within the total population. Φ_CT_−proportion of the total variance explained by the grouping.
(PDF)
