# Supplementary material for: Winter temperature correlates with mtDNA genetic structure of yellow-necked mouse population in NE Poland
Source: PLoS One. 2019 May 8;14(5):e0216361. doi: 10.1371/journal.pone.0216361 (PMC6505929; doi:10.1371/journal.pone.0216361)
Supplement: S1 Table — Numbers of identified mtDNA haplotypes that belong to each defined mtDNA haplogroup. (DOCX) [file pone.0216361.s001.docx]

**Supporting Information**

S1 Table. Number of samples successfully analysed per geographical region (mtDNA). Numbers of identified mtDNA haplotypes that belong to each defined mtDNA haplogroup.

|  |  | | | | |
| --- | --- | --- | --- | --- | --- |
| **Region** | **mtDNA** | **Haplogroup 1** | **Haplogroup 2** | **Haplogroup 3** |  |
| Augustów Forest 2006 | – | – | – | – |  |
| Augustów Forest 2007 | 16 | 1 | 8 | 7 |  |
| Augustów Forest 2008 | 8 | 0 | 7 | 1 |  |
| Białowieża Forest 2004 | 2 | 2 | 0 | 0 |  |
| Białowieża Forest 2005 | 7 | 2 | 5 | 0 |  |
| Białowieża Forest 2007 | 56 | 39 | 5 | 12 |  |
| Białowieża Forest 2008 | 21 | 16 | 3 | 2 |  |
| Borki Forest 2004 | 11 | 5 | 1 | 5 |  |
| Borki Forest 2006 | 3 | 1 | 1 | 1 |  |
| Knyszyn Forest 2004 | 1 | 1 | 0 | 0 |  |
| Knyszyn Forest 2006 | 10 | 2 | 3 | 5 |  |
| Knyszyn Forest 2007 | 15 | 3 | 5 | 7 |  |
| Knyszyn Forest 2008 | 5 | 1 | 4 | 0 |  |
| Mielnik Forest 2005 | 12 | 2 | 5 | 5 |  |
| Mielnik Forest 2006 | – | – | – | – |  |
| Mielnik Forest 2007 | 31 | 16 | 9 | 6 |  |
| Mielnik Forest 2008 | 34 | 15 | 9 | 10 |  |
| Pisz Forest 2004 | 9 | 2 | 0 | 7 |  |
| Rominta Forest 2004 | 20 | 2 | 5 | 13 |  |
| Rominta Forest 2006 | 10 | 2 | 1 | 7 |  |
| Aug – Knysz Transect 2007 | 40 | 0 | 17 | 23 |  |
| Aug – Knysz Transect 2008 | 19 | 7 | 3 | 9 |  |
| Knysz – Bial Transect 2007 | 20 | 11 | 1 | 8 |  |
| Knysz – Bial Transect 2008 | – | – | – | – |  |
| Bial – Miel Transect 2007 | 3 | 1 | 0 | 2 |  |
| Bial – Miel Transect 2008 | – | – | – | – |  |
| Total | 353 | 131 | 92 | 130 |  |
