# Supplementary material for: Winter temperature correlates with mtDNA genetic structure of yellow-necked mouse population in NE Poland
Source: PLoS One. 2019 May 8;14(5):e0216361. doi: 10.1371/journal.pone.0216361 (PMC6505929; doi:10.1371/journal.pone.0216361)
Supplement: S2 Table — (DOCX) [file pone.0216361.s002.docx]

S2 Table. Yellow–necked mouse cyt *b* mtDNA sequences available at NCBI GenBank used for comparison with material from this study. Sequences that were identified as identical to any of 247 bp haplotypes found in this study have assigned haplotype number and the haplogroup number (in brackets)

| Accession  number | Haplotype  NE Poland  (clade) | Location | Source |
| --- | --- | --- | --- |
| JF819962.1 | H2 (3) | Macedonia | Krystufek et al. [1] |
| JF819964 | H2 (3) | Macedonia | ” ” |
| AJ605641.1 | H2 (3) | Lithuania, Moretz | Michaux et al. [2] |
| AJ605610.1 | H2 (3) | Estonia, Tallin | ” ” |
| AJ605602.1 | H2 (3) | Belarus, Berezina | ” ” |
| AJ605614.1 | H2 (3) | France, Correze | ” ” |
| AJ605651.1 | H2 (3) | Russia, Samara | ” ” |
| AJ605650.1 | H2 (3) | Russia, Samara | ” ” |
| AJ605603.1 | H5 (2) | Belarus, Berezina | Michaux et al. [2] |
| KC676646.1 | H6 (1) | Croatia | Svoboda et al. [3] |
| KC676643.1 | H6 (1) | Croatia | ” ” |
| AY179501.1 | H6 (1) | Switzerland: Aclens | Reutter et al. [4] |
| AY158453.1 | H6 (1) | Ukraine: Chornobyl | Dunina–Barkovskaya et al. [5] |
| AJ631970.1 | H7 (1) | Sweden, Golland | Michaux et al. [2] |
| AJ605616.1 | H7 (1) | Germany, Bielefeld | ” ” |
| AJ605686.1 | H7 (1) | Ukraine, Southern Bug | ” ” |
| AJ605627.1 | H7 (1) | Greece, Peloponnese | ” ” |
| AJ605636.1 | H7 (1) | Italy, Aspromonte | ” ” |
| AJ605634.1 | H7 (1) | Hungary, Debrecen | ” ” |
| AJ605631.1 | H7 (1) | Greece, Macedonia | ” ” |
| AJ605626.1 | H7 (1) | Greece, Peloponnese | ” ” |
| AJ605625.1 | H7 (1) | Greece, Peloponnese | ” ” |
| AJ605624.1 | H7 (1) | Greece, Peloponnese | ” ” |
| AJ605623.1 | H7 (1) | Greece, Mt. Olympus | ” ” |
| AJ605622.1 | H7 (1) | Greece, Mt. Olympus | ” ” |
| AJ605621.1 | H7 (1) | Greece, Mt. Olympus | ” ” |
| AJ605620.1 | H7 (1) | Greece, Mt. Olympus | ” ” |
| AJ605642.1 | H7 (1) | Macedonia, Bistra | ” ” |
| AJ605615.1 | H7 (1) | France, Py Mantet | ” ” |
| AJ605666.1 | H7 (1) | Turkey:Damar | ” ” |
| AJ605664.1 | H7 (1) | Sweden | ” ” |
| AJ605663.1 | H7 (1) | Sweden | ” ” |
| AJ605663.1 | H7 (1) | Sweden | ” ” |
| AJ605657.1 | H7 (1) | Slovenia, Asan cesma | ” ” |
|  |  |  | Continued on the next page |
| Table S2. – continued. | | | |
| AJ605605.1 | H7 (1) | Czech Republic | Michaux et al. [2] |
| AJ605675.1 | H7 (1) | Turkey, Thrace | ” ” |
| AJ605674.1 | H7 (1) | Turkey, Thrace | ” ” |
| AJ605600.1 | H7 (1) | Austria, Vorhalberg | ” ” |
| AJ298603.1 | H7 (1) | Germany, Bielefeld | ” ” |
| DQ389586.1 | H7 (1) | Germany, Bavaria | Essbauer et al. (unpub.) (2006?) |
| DQ389585.1 | H7 (1) | Germany, Bavaria | ” ” |
| DQ389584.1 | H7 (1) | Germany, Bavaria | ” ” |
| DQ389583.1 | H7 (1) | Germany, Bavaria | ” ” |
| DQ389582.1 | H7 (1) | Germany, Bavaria | ” ” |
| DQ389594.1 | H7 (1) | Germany, Bavaria | ” ” |
| DQ389593.1 | H7 (1) | Germany, Bavaria | ” ” |
| DQ389590.1 | H7 (1) | Germany, Bavaria | ” ” |
| DQ389592.1 | H7 (1) | Germany, Bavaria | ” ” |
| DQ389588.1 | H7 (1) | Germany, Bavaria | ” ” |
| DQ389598.1 | H7 (1) | Germany, Bavaria | ” ” |
| DQ389597.1 | H7 (1) | Germany, Bavaria | ” ” |
| JF819968.1 | H7 (1) | Bosnia and Herzegovina | Krystufek et al. [1] |
| JF819967.1 | H7 (1) | Greece | ” ” |
| JF819959.1 | H7 (1) | Macedonia | ” ” |
| JF819961.1 | H7 (1) | Macedonia | ” ” |
| JF819958.1 | H7 (1) | Macedonia | ” ” |
| JX457728.1 | H7 (1) | France | Barbosa et al. [6] |
| AB032853.1 | H7 (1) | Switzerland | Serizawa et al. [7] |
| AY179498.1 | H7 (1) | Germany, Feldsee | Reutter et al. [4] |
| KC676638.1 | H7 (1) | Croatia | Svoboda et al. [3] |
| KC676648.1 | H7 (1) | Croatia | ” ” |
| GU060534.1 | H7 (1) | Germany | Guenther et al. (unpub.) |
| GU060534.1 | H7 (1) | Germany | ” ” |
| AY158452.1 | H7 (1) | Ukraine, Chornobyl | Dunina–Barkovskaya et al. [5] |
| AY158445.1 | H7 (1) | Ukraine, Chornobyl | ” ” |
| AY158443.1 | H7 (1) | Ukraine, Chornobyl | ” ” |
| AY158454.1 | H10 (3) | Ukraine, Chornobyl | Dunina–Barkovskaya et al. [5] |
| AJ605601.1 | H10 (3) | Belarus, Berezina | Michaux et al. [2] |
| AY158449.1 | H20 (3) | Ukraine, Chornobyl | Dunina–Barkovskaya et al. [5] |
| AY158447.1 | H20 (3) | Ukraine, Chornobyl | ” ” |
| AY158446.1 | H20 (3) | Ukraine, Chornobyl | ” ” |
| AY158444.1 | H20 (3) | Ukraine, Chornobyl | Dunina–Barkovskaya et al. [5] |
| AF159392.1 |  | Germany, Konstanz | Martin et al. [8] |
| JF819963.1 |  | Macedonia | Krystufek et al. [1] |
| AY179502.1 |  | Switzerland | Reutter et al. [4] |
| AJ605609.1 |  | Czech Republic | Michaux et al. [2] |
|  |  |  | Continued on the next page |
| Table S2. – continued. | | | |
| AJ605632.1 |  | Greece, Epirus | Michaux et al. [2] |
| AJ605670.1 |  | Turkey, Egridir | ” ” |
| AJ605668.1 |  | Turkey, Datca | ” ” |
| AJ605653.1 |  | Russia, Volgograd | ” ” |
| AJ605652.1 |  | Russia, Volgograd | ” ” |
| AJ605648.1 |  | Romania | ” ” |
| AJ605647.1 |  | Romania | ” ” |
| AJ605692 |  | Yugoslavia, Susarra | ” ” |
| AJ605691 |  | Yugoslavia, Susarra | ” ” |
| AJ605646.1 |  | Romania | ” ” |
| AY158451.1 |  | Ukraine, Chornobyl | Dunina–Barkovskaya et al. [5] |
| AY179499.1 |  | Germany, Feldsee | Reutter et al. [4] |
| AJ605637 |  | Italy, Aspromonte | Michaux et al. [2] |
| AJ605640.1 |  | Italy, Abruzzo | ” ” |
| AJ298604.1 |  | Italy, Aspromonte | Michaux et al. [9] |
| AJ311150 |  | Italy, Abruzzo | Michaux et al. [10] |
| AJ631969.1 |  | Spain, Navarra | Michaux et al. [2] |
| AJ605661.1 |  | Spain, Navarra | ” ” |
| AJ605660.1 |  | Spain, Navarra | ” ” |
| AJ605690.1 |  | Israel, Mt. Carmel | ” ” |
| AJ605608.1 |  | Czech Republic | ” ” |
| AJ605689 |  | Israel, Mt. Carmel | ” ” |
| AJ605688 |  | Iran:Horamabad | ” ” |
| AJ605687.1 |  | Iran:Horamabad | ” ” |
| AJ605667.1 |  | Turkey, Datca | ” ” |
| AJ605669 |  | Turkey, Egridir | ” ” |
| AJ605635.1 |  | Italy, Grosseto | ” ” |
| AJ605633.1 |  | Greece, Epirus | ” ” |
| AJ605629.1 |  | Greece, Macedonia | ” ” |
| DQ379300.1 |  | Germany | Splettstoesser et al*.* [11] |
| AJ605628.1 |  | Greece, Peloponnese | Michaux et al. [2] |
| AJ605618.1 |  | Greece, Mt. Olympus | ” ” |
| AJ605619.1 |  | Greece, Mt. Olympus | ” ” |
| AJ605617.1 |  | Greece, Mt. Olympus | ” ” |
| AJ605613.1 |  | France, Allier | ” ” |
| AY179500.1 |  | Switzerland, Gollion | Reutter et al. [4] |
| AJ605612.1 |  | France, Allier | Michaux et al. [2] |
| AJ298602.1 |  | France, Allier | ” ” |
| AJ605611.1 |  | Estonia, Tallin | Michaux et al. [9] |
| AJ605644.1 |  | Macedonia, Bistra | Michaux et al. [2] |
|  |  |  | Continued on the next page |
| Table S2. – concluded. | | |  |
| AJ605643.1 |  | Macedonia, Bistra | Michaux et al. [2] |
| JF819955.1 |  | Macedonia | Krystufek et al. [1] |
| JF819969.1 |  | Bosnia and Herzegovina | ” ” |
| AJ605606.1 |  | Czech Republic | Michaux et al. [2] |
| JX457730.1 |  | Spain, Great Britain, Poland, Finland | Barbosa et al. [6] |
| JX457732.1 |  | Poland | ” ” |
| JX457729.1 |  | France, Poland | ” ” |
| GQ260185.1 |  | Germany | Schlegel et al. [12] |
| JF819957.1 |  | Macedonia | Krystufek et al. [1] |
| AY158450.1 |  | Ukraine: Chornobyl | Dunina–Barkovskaya et al. [5] |
| AJ605672 |  | Turkey:Thrace | Michaux et al. [2] |
| DQ090754.1 |  | Germany: Bavaria | Essbauer et al. (unpub.) |
| KC676639.1 |  | Croatia | Svoboda et al. 2014 |
| AJ605677.1 |  | Turkey | Michaux et al. [2] |
| AJ605676.1 |  | Turkey | ” ” |
| AJ605654.1 |  | Russia:Voronezh | Michaux et al. [2] |
| AJ298601.1 |  | Belgium:Gembes | Michaux et al. [2] |
| AJ311151.1 |  | France:Allier | ” ” |
| FJ389660.1 |  | Germany | Dubey et al. [13] |
| JX457731.1 |  | Spain, Poland | Barbosa et al. [6] |
| JX457727.1 |  | Great Britain | Barbosa et al. [6] |
| JF819966.1 |  | Bosnia and Herzegovina | Krystufek et al. [1] |
| JF819965.1 |  | Bosnia and Herzegovina | Krystufek et al. [1] |
| JF819960.1 |  | Macedonia | Krystufek et al. [1] |
| JF819956.1 |  | Macedonia | Krystufek et al. [1] |
| KC676645.1 |  | Croatia | Svoboda et al. [3] |
| KC676644.1 |  | Croatia | Svoboda et al. [3] |
| AJ605656.1 |  | Slovenia:Skofja | Michaux et al. [2] |
| KC676642.1 |  | Croatia | Svoboda et al. [3] |
| KC676641.1 |  | Croatia | Svoboda et al. [3] |
| JF819970.1 |  | Bosnia and Herzegovina | Krystufek et al. [1] |
| KC676640.1 |  | Croatia | Svoboda et al. [3] |
| KC676637.1 |  | Croatia | Svoboda et al. [3] |
| KC676636.1 |  | Croatia | Svoboda et al. [3] |
| DQ389595.1 |  | Germany | Essbauer et al.(unpub.) |
| DQ389589.1 |  | Germany | ” ” |
| DQ389587.1 |  | Germany | ” ” |
| DQ389579.1 |  | Germany | Essbauer et al. (unpub.) |
| GQ260185.1 |  | Germany | Schlegel et al. [12] |

**References**

1. Kryštufek B, Luznik M, Buzan EV. Mitochondrial cytochrome b sequences resolve the taxonomy of field mice (Apodemus) in the western Balkan refugium. Acta Theriologica. 2012 Jan;57(1):1-7.
2. Michaux JR, Libois R, Paradis E, Filippucci MG. Phylogeographic history of the yellow-necked fieldmouse (Apodemus flavicollis) in Europe and in the Near and Middle East. Molecular Phylogenetics and Evolution. 2004 Sep;32(3):788-98.
3. Svoboda P, Dobler G, Markotic A, Kurolt IC, Speck S, Habus J, et al. Survey for Hantaviruses, Tick-Borne Encephalitis Virus, and Rickettsia spp. in Small Rodents in Croatia. Vector-Borne and Zoonotic Diseases. 2014 Jul;14(7): 523-30.
4. Reutter BA, Petit E, Brunner H, Vogel P. Cytochrome b haplotype divergences in West European Apodemus. Mamm Biol. 2003;68(3):153-64.
5. Dunina-Barkovskaya YV. Population genetics of rodents living in the Chornobyl environment based on mitochondrial and nuclear gene sequences. Unpublished M.S. thesis, Texas Tech University, Lubbock; 2004. Available from: <https://ttu-ir.tdl.org/ttuir/bitstream/handle/2346/10867/31295019458529.pdf?sequence=1>.
6. Barbosa S, Pauperio J, Searle JB, Alves PC. Genetic identification of Iberian rodent species using both mitochondrial and nuclear loci: application to noninvasive sampling. Mol Ecol Resour. 2013 Jan;13(1):43-56.
7. Serizawa K, Suzuki H, Tsuchiya K. A phylogenetic view on species radiation in Apodemus inferred from variation of nuclear and mitochondrial genes. Biochemical Genetics. 2000 Feb;38(1-2):27-40.
8. Martin Y, Gerlach G, Schlotterer C, Meyer A. Molecular phylogeny of European muroid rodents based on complete cytochrome b sequences. Molecular Phylogenetics and Evolution. 2000 Jul;16(1):37-47.
9. Michaux JR, Kinet S, Filippucci MG, Libois R, Besnard A, Catzeflis F. Molecular identification of three sympatric species of wood mice (Apodemus sylvaticus, A-flavicollis, A-alpicola) in western Europe (Muridae: Rodentia). Molecular Ecology Notes. 2001 Dec;1(4):260-3.
10. Michaux JR, Chevret P, Filippucci MG, Macholan M. Phylogeny of the genus Apodemus with a special emphasis on the subgenus Sylvaemus using the nuclear IRBP gene and two mitochondrial markers: cytochrome b and 12S rRNA. Molecular Phylogenetics and Evolution. 2002 May;23(2):123-36.
11. Splettstoesser WD, Matz-Rensing K, Seibold E, Tomaso H, Al. Dahouk S, Grunow R,

Essbauer S, Buckendahl A, Finke EJ and Neubauer H. Re-emergence of Francisella tularensis in Germany: fatal tularaemia in a colony of semi-free-living marmosets (Callithrix jacchus). Epidemiol. Infect. 2007 135(8):1256-1265.

1. Schlegel M, Klempa B, Auste B, Bemmann M, Schmidt-Chanasit J, Buchner T,

Groschup MH, Meier M, Balkema-Buschmann A, Zoller H, Kruger DH, Ulrich RG.

Dobrava-belgrade virus spillover infections, Germany. Emerging Infect. Dis. 2009 15: 2017-2020.

1. Dubey S, Michaux J, Brunner H, Hutterer R, Vogel P. False phylogenies on wood mice due to cryptic cytochrome-b pseudogene. Molecular Phylogenetics and Evolution. 2009 Mar;50(3):633-41.
