# Supplementary material for: Winter temperature correlates with mtDNA genetic structure of yellow-necked mouse population in NE Poland
Source: PLoS One. 2019 May 8;14(5):e0216361. doi: 10.1371/journal.pone.0216361 (PMC6505929; doi:10.1371/journal.pone.0216361)
Supplement: S3 Table — The numbers of haplogroup of each of the haplotypes are in brackets. (DOCX) [file pone.0216361.s003.docx]

S3 Table. Distribution and number of cyt b mtDNA haplotypes detected in samples collected in seven forests and three transects: ROM – Rominta Forest, BOR – Borki, PIS – Pisz, AUG – Augustów, BIAŁ – Białowieża, MIEL – Mielnik, TAK – Augustów-Knyszyn Transect, TKB– Knyszyn-Białowieża, TBM – Białowieża-Mielnik. The numbers of haplogroup of each of the haplotypes are in brackets.

| Haplotype  (haplogroup) | ROM | BOR | PIS | AUG | TAK | KNYSZ | TKB | BIAŁ | TBM | MIEL | Total  N (%) |
| --- | --- | --- | --- | --- | --- | --- | --- | --- | --- | --- | --- |
| H21 (3) | 5 | – | – | – | – | – | – | – | – | – | 5 (1.4) |
| H20 (3) | 5 | – | – | – | – | – | – | – | – | – | 5 (1.4) |
| H19 (3) | 3 | – | – | – | – | – | – | – | – | – | 3 (0.8) |
| H18 (2) | 6 | 1 | – | – | – | – | – | – | – | – | 7 (2.0) |
| H13 (1) | – | 1 | – | – | – | – | – | – | – | – | 1 (0.3) |
| H14 (2) | – | 1 | – | – | – | – | – | – | – | – | 1 (0.3) |
| H17 (3) | – | 5 | – | – | – | – | – | – | – | 1 | 6 (1.7) |
| H24 (1) | – | – | 1 | – | – | – | – | – | – | – | 1 (0.3) |
| H22 (1) | – | – | 1 | – | – | – | – | 1 | – | – | 2 (0.6) |
| H2 (3) | – | – | 1 | 2 | 7 | 1 | – | 1 | – | 6 | 18 (5.1) |
| H1 (3) | – | – | 3 | 4 | 1 | – | – | – | – | – | 8 (2.3) |
| H16 (3) | 1 | – | 3 | – | 4 | 1 | – | – | – | – | 9 (2.5) |

S3 Table – continued

| Haplotype  (haplogroup) | ROM | BOR | PIS | AUG | TAK | KNYSZ | TKB | BIAŁ | TBM | MIEL | Total  N (%) |
| --- | --- | --- | --- | --- | --- | --- | --- | --- | --- | --- | --- |
| H10 (3) | 5 | 1 | – | – | 4 | 1 | – | 2 | 1 | – | 14 (4.0) |
| H7 (1) | 4 | 5 | – | 1 | 6 | 5 | 4 | 47 | – | 12 | 84 (23.8) |
| H3 (3) | 1 | – | – | 2 | 14 | 8 | 8 | 11 | 1 | 13 | 58 (16.4) |
| H4 (2) | – | – | – | 7 | 9 | 2 | – | – | – | 2 | 20 (5.7) |
| H5 (2) | – | – | – | 8 | 11 | 7 | – | 11 | – | 13 | 50 (14.2) |
| H9 (3) | – | – | – | – | 2 | – | – | – | – | 1 | 3 (0.8) |
| H11 (1) | – | – | – | – | 1 | – | 2 | 2 | – | 2 | 7 (2.0) |
| H12 (2) | – | – | – | – | – | 4 | 1 | 2 | – | 5 | 12 (3.4) |
| H6 (1) | – | – | – | – | – | 1 | 5 | 9 | 1 | 18 | 34 (9.6) |
| H15 (3) | – | – | – | – | – | 1 | – | – | – | – | 1 (0.3) |
| H8 (2) | – | – | – | – | – | – | – | – | – | 3 | 3 (0.8) |
| H23 (2) | – | – | – | – | – | – | – | – | – | 1 | 1 (0.3) |
| All | 30 | 14 | 9 | 24 | 59 | 31 | 20 | 86 | 3 | 77 | 353 (100) |
| Haplogroup1  Haplogroup 2 | 4  6 | 6  2 | 2  – | 1  15 | 7  20 | 6  13 | 11  1 | 59  13 | 1  – | 32  24 | 129 (36)  94 (27) |
| Haplogroup 3 | 20 | 6 | 7 | 8 | 32 | 12 | 8 | 14 | 2 | 21 | 130 (37) |
