# Supplementary material for: Winter temperature correlates with mtDNA genetic structure of yellow-necked mouse population in NE Poland
Source: PLoS One. 2019 May 8;14(5):e0216361. doi: 10.1371/journal.pone.0216361 (PMC6505929; doi:10.1371/journal.pone.0216361)
Supplement: S4 Table — See Table 1 for abbreviations of regions and Fig 2 for their location. Percentage of land use categories based on Corine Landcover 2006 (CLC2006) data in 1-km buffer zone around each trapping site. Mean temperature collected based on four measurements per month of land surface temperature in a spatial grid 1 km × 1 km (MODIS). (DOCX) [file pone.0216361.s004.docx]

S4 Table Ecological characteristics of the studied forests and transects and abundance indices of yellow-necked mice. See Table 1 for abbreviations of regions and Fig. 2 for their location. Percentage of land use categories based on Corine Landcover 2006

(CLC2006) data in 1-km buffer zone around each trapping site. Mean temperature collected based on four measurements per month of land surface temperature in a spatial grid 1 km × 1 km (MODIS).

| Region | Mean temperature (°C) | |  | | Percentage cover of land use category | | | | | | |  |
| --- | --- | --- | --- | --- | --- | --- | --- | --- | --- | --- | --- | --- |
|  | January | July |  | Coniferous  forest | | Mixed forest | Deciduous  forest | Meadows and arable land | Waters | | Other | |
| AUG | –5.26 | 22.52 |  | 50.5 | | 31.3 | 4.0 | 7.5 | 3.5 | | 3.2 | |
| BIAL | –2.74 | 20.71 |  | 29.6 | | 27.9 | 41.3 | 1.2 | 0 | | 0 | |
| BOR | –6.80 | 21.87 |  | 15.1 | | 63.4 | 12.4 | 0.6 | 6.8 | | 1.7 | |
| KNYSZ | –4.71 | 22.38 |  | 58.4 | | 16.9 | 8.3 | 12.2 | 0 | | 4.2 | |
| MIEL | –9.85 | 19.93 |  | 24.3 | | 5.6 | 65.4 | 2.8 | 0 | | 1.9 | |
| ROM | –11.40 | 20.58 |  | 16.4 | | 48.0 | 25.5 | 7.4 | 0 | | 2.7 | |
| PIS | –6.79 | 21.33 |  | 46.0 | | 26.5 | 15.8 | 7.9 | 1.7 | | 2.1 | |
| Mean for Forests | –6.79 | 21.33 |  | 34.3 | | 31.4 | 24.7 | 5.6 | 1.7 | | 2.3 | |
| TAK | –6.41 | 22.79 |  | 18.4 | | 11.1 | 25.4 | 42.5 | 0 | | 2.6 | |
| TKB | –8.43 | 22.35 |  | 59.4 | | 2.3 | 8.0 | 26,2 | 1.4 | | 2.7 | |
| TBM | –7.24 | 22.24 |  | 53.4 | | 5.6 | 3.3 | 25.8 | 0 | | 11.9 | |
| Mean for Transects | –7.36 | 22.46 |  | 43.7 | | 6.3 | 12.2 | 31.5 | 0.5 | 5.7 | | |
