# Supplementary material for: Winter temperature correlates with mtDNA genetic structure of yellow-necked mouse population in NE Poland
Source: PLoS One. 2019 May 8;14(5):e0216361. doi: 10.1371/journal.pone.0216361 (PMC6505929; doi:10.1371/journal.pone.0216361)
Supplement: S1 Fig — (PDF) [file pone.0216361.s005.pdf]

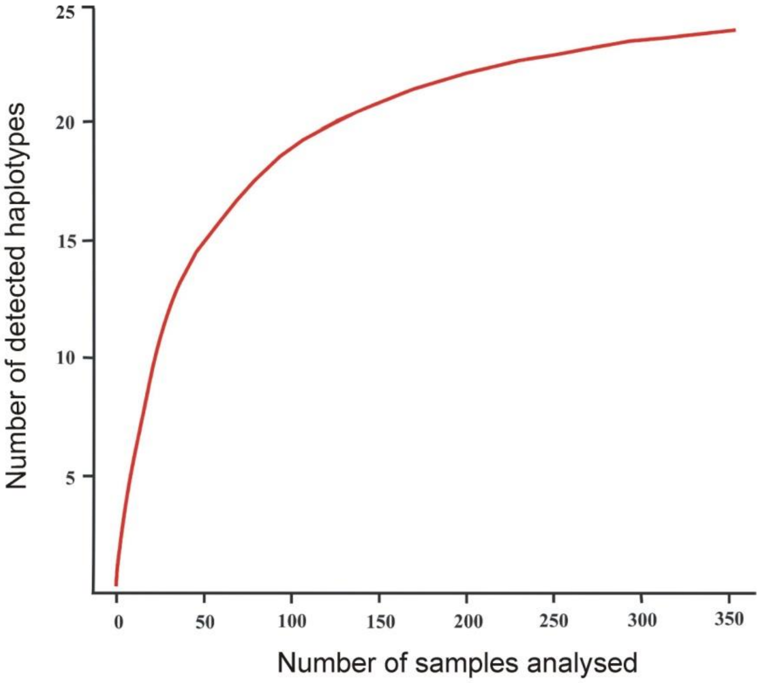

S1 Fig. The rarefaction curve presenting a dependence of number of detected haplotypes on a number of analysed samples.
