# Supplementary material for: Winter temperature correlates with mtDNA genetic structure of yellow-necked mouse population in NE Poland
Source: PLoS One. 2019 May 8;14(5):e0216361. doi: 10.1371/journal.pone.0216361 (PMC6505929; doi:10.1371/journal.pone.0216361)
Supplement: S2 Fig — ΦSC−proportion of the variance among local populations within groups. ΦST−proportion of the variance among local populations within the total population. ΦCT−proportion of the total variance explained by the grouping. (PDF) [file pone.0216361.s006.pdf]

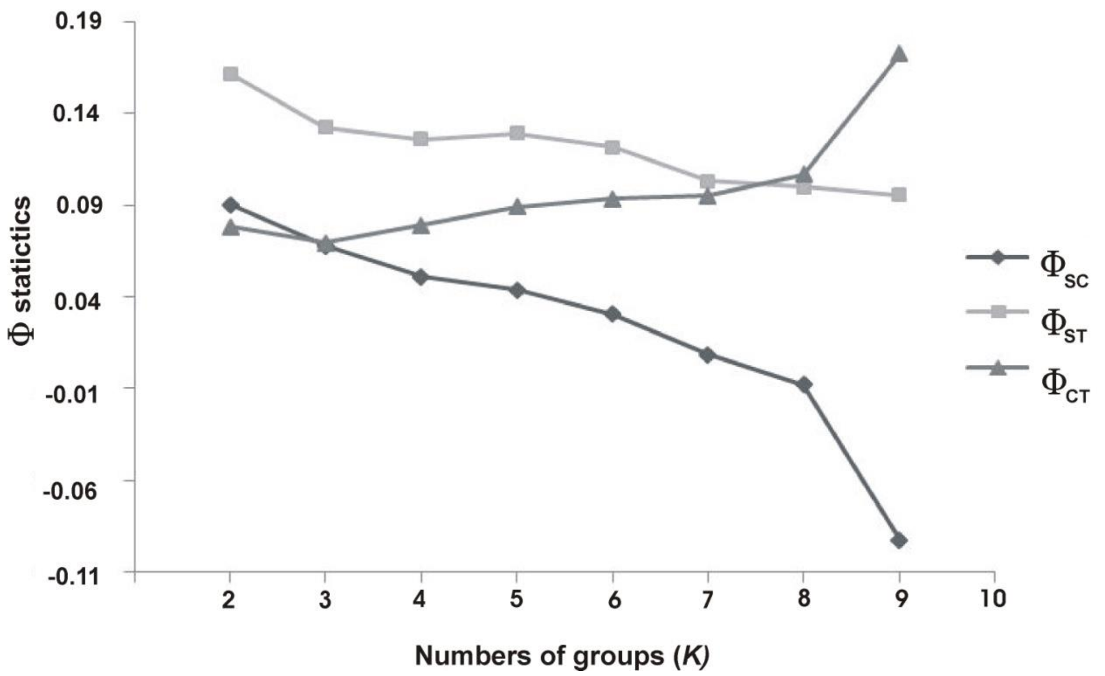

S2 Fig. Changes in Φ statistics for K = 2 to 9 subpopulations of yellow–necked mice in north-eastern Poland, on the basis of mtDNA and inferred from SAMOVA. Φ_{SC} – proportion of the variance among local populations within groups. Φ_{ST} – proportion of the variance among local populations within the total population. Φ_{CT} – proportion of the total variance explained by the grouping.
